# CLDN6 induces chemoresistance through protective autophagy in breast cancer

**DOI:** 10.7150/ijbs.116340

**Published:** 2025-08-22

**Authors:** Huinan Qu, Qiu Jin, Mingzi Zhang, Da Qi, Minghao Sun, Yuan Dong, Chengshi Quan

**Affiliations:** 1 The Key Laboratory of Pathobiology, Ministry of Education, College of Basic Medical Sciences, Jilin University, 126 Xinmin Avenue, Changchun, Jilin 130021, China.; 2 Department of Histology and Embryology, College of Basic Medical Sciences, Jilin University, 126 Xinmin Avenue, Changchun, Jilin 130021, China.; 3 The Zilkha Neurogenetic Institute Department of Physiology and Neuroscience Keck School of Medicine, University of Southern California, Los Angeles, CA 90033, USA.

**Keywords:** CLDN6, LKB1, protective autophagy, chemoresistance, breast cancer

## Abstract

Protective autophagy, a defensive response of cancer cells to chemotherapeutic stress, plays a critical role in the development of chemoresistance. Our previous research has demonstrated that the tight junction protein Claudin-6 (CLDN6) can induce autophagy and chemoresistance respectively. However, it remains unclear whether CLDN6 triggers protective autophagy under chemotherapeutic conditions. In this study, we focused on the role and mechanism of CLDN6 in inducing protective autophagy and promoting chemoresistance in breast cancer. We found that CLDN6 promoted chemoresistance by inducing protective autophagy in response to adriamycin (ADM) and paclitaxel (PTX). Mechanistically, CLDN6 interacted with LKB1 through its PDZ-binding motif, leading to the activation of AMPK/ULK1 signaling and subsequent promotion of protective autophagy. Notably, we discovered that chemotherapy increased CLDN6 expression through the reactive oxygen species (ROS)/GATA4 axis. Our results suggest that CLDN6 plays a pivotal role in breast cancer chemoresistance through protective autophagy, highlighting its potential as a therapeutic target to improve treatment outcomes of breast cancer patients.

## Introduction

Despite the emergence of new chemotherapeutic agents, tumor cells can develop chemoresistance through various mechanisms, which is one of the major causes of recurrence and advanced metastasis in breast cancer patients 1. Understanding the mechanisms underlying chemoresistance is critical to managing breast cancer more effectively and improving patient survival rates.

Autophagy is a lysosome-dependent self-digestive process for catabolism of proteins and damaged organelles. Recent studies have demonstrated that autophagy induced by chemotherapy is associated with chemoresistance in various types of cancer 2, 3. It is currently considered that cancer cells can induce at least two forms of autophagy, namely protective autophagy and non-protective autophagy, with only protective autophagy conferring resistance to chemotherapy 4. However, protective and non-protective autophagy can't be distinguished based on morphological and biochemical characteristics 5. Therefore, identifying biomarkers associated with protective autophagy is of significant clinical importance.

CLDNs, a major component of cellular tight junctions, are primarily located on the surface of epithelial cells, where they perform barrier and fenestration functions 6. Our previous research revealed that CLDN6 is expressed at low levels in breast cancer, while its overexpression inhibits breast cancer growth and metastasis. Furthermore, this inhibitory effect on growth and metastasis is associated with CLDN6-mediated non-protective autophagy 7-10. Notably, CLDN6 is highly expressed in multidrug-resistant breast cancer cells MCF-7/MDR and promotes chemoresistance to ADM, 5-fluorouracil, and cisplatin 11. It appears that CLDN6 has a “pro-oncogenic” effect under chemotherapy conditions, contradicting its cancer-suppressive role in breast cancer. Importantly, we found that high expression of CLDN6 is associated with high levels of autophagy in MCF-7/MDR cells via preliminary experiments. However, existing research has not yet clarified whether CLDN6 promotes chemoresistance by inducing protective autophagy under chemotherapy conditions; the mechanism by which CLDN6 induces protective autophagy; and whether the “pro-oncogenic” role of CLDN6 under chemotherapy conditions depends on its abnormal expression and localization.

Unc-51 Like Autophagy Activating Kinase 1 (ULK1), a crucial protein involved in the initiation of autophagy, regulating the localization and cascade reactions of autophagy-related proteins (ATGs) on various autophagosome membranes 12. Numerous studies indicate that ULK1-induced autophagy plays a role in regulating cancer growth, metastasis, and chemoresistance 13-15. Our previous mRNA sequencing of MCF-7/CLDN6 cells showed significant alterations in the expression of 15 autophagy-related genes, including ULK1. As a protein with serine/threonine kinase activity, the Ser555 site of ULK1 can be phosphorylated by AMPK to initiate autophagy 16, 17. Liver kinase B1 (LKB1), a key upstream regulator of AMPK, can induce autophagy by activating AMPK/ULK1 signaling 18, 19. Therefore, we hypothesized that CLDN6 might induce protective autophagy via LKB1/AMPK/ULK1 signaling under chemotherapeutic conditions.

Chemotherapeutic agents can elevate intracellular ROS levels, which function as signaling molecules to enhance the transcription of genes such as GATA4 20, 21. GATA4 is a member of the GATA family of zinc-finger transcription factors 22, and was identified as a transcription factor for CLDN6 based on predictions from databases such as hTFtarget and knockTF. Therefore, we hypothesized that chemotherapy may upregulate CLDN6 expression in breast cancer cells through the ROS/GATA4 axis.

In the present study, we first assessed the relationship between CLDN6 expression and patient survival after chemotherapy in breast cancer patient tissues. Next, we used *in vitro* and *in vivo* experiments to confirm whether CLDN6 promotes chemoresistance through protective autophagy and further explored its molecular mechanisms. Finally, we investigated the effects of chemotherapy on CLDN6 expression and localization and the possible pathways involved. This study aims to validate the potential of CLDN6 as a biomarker for protective autophagy, providing a novel therapeutic approach for chemoresistant breast cancer patients.

## Materials and Methods

### Predictive value analysis based on ROC plotter

The ROC plotter was used to evaluate the relationship between CLDN6 expression and response to a specific therapy in November 2020. Briefly, this tool links CLDN6 expression to therapy response using transcriptomic data from 3104 breast tumor samples. Treatment options included anthracycline and taxane. Treatment response was identified based on pathological complete response (pCR) and relapse-free survival (RFS) at 5 years. The responder group refers to patients who achieved pCR or RFS at 5 years after receiving specific treatment, while those who did not achieve this are classified as the non-responder group. A box plot displaying CLDN6 expression levels in the responder group and non-responder group is provided along with the area under the curve (AUC) and their respective *P* values.

### Immunohistochemistry (IHC)

Human breast cancer paraffin tissue microarray (TMA; AF-BrcSur2202) was purchased from Aifang Biotechnology (Hunan, China), with ethics approval number HN20250401, approved by the Ethics Review Committee for Biological Sciences and Technology of Hunan Aifang Biotechnology Co., Ltd. The TMA comprises 36 specimens from triple-negative breast cancer (TNBC) patients who received surgery following neoadjuvant chemotherapy. Exclusion criteria were patients receiving targeted therapy, traditional Chinese medicine treatment, or adjuvant chemotherapy. The paraffin TMA was dehydrated in xylene, anhydrous ethanol, 95% ethanol, and 85% ethanol and then subjected to antigen repair. Subsequently, the sections were treated with the UltraSensitive SP (Mouse/Rabbit) IHC Kit (KIT-9710, MXB Biotechnology, Fuzhou, China). The expression level of CLDN6 was quantified using the H-score of the staining signal. H-score = (percentage of cells of weak intensity × 1) + (percentage of cells of moderate intensity × 2) + (percentage of cells of strong intensity × 3). Survival analysis groupings were divided into high and low expression groups based on the median total score.

### Cell culture

MCF-7, MDA-MB-231, MCF-7/MDR, and human embryonic kidney (HEK) 293T cells were cultured in Dulbecco's Modified Eagle's Medium (DMEM; Meilune, China) supplemented with 10% fetal bovine serum (FBS; Gibco, USA) at 37℃ in a humidified incubator containing 5% CO2. The MCF-7 (cat. no. ZQ0071) and MDA-MB-231 (cat. no. ZQ0118), were procured from Zhong Qiao Xin Zhou Biotechnology (Shanghai, China).

### Antibodies and reagents

Details of the antibodies and reagents used in this study are shown in Supplementary Table S1.

### Transfection

The ULK1 shRNA plasmid and AMPK shRNA plasmid were produced by PPL Genebio Technology (Nanjing, China). The CLDN6-GFP-luciferase overexpression and the CLDN6 mt (the PDZ-binding motif was deleted) overexpression lentivirus were constructed by Genechem (Shanghai, China). The GATA4 overexpression lentivirus was generated by Fenghui Biotechnology (Changsha, China). The transfection process was conducted as described by Yang et al. 11.

### Western blot (WB)

WB assay was performed as previously described 8.

### Cell Counting Kit 8 (CCK8) assay

Cells were cultured in 96-well plates (5 × 10^3^ cells per well). Following adhesion, the cells were pretreated with the autophagy inhibitor chloroquine (CQ) 10 µM for 24 hours as necessary. Subsequently, the cells were treated with ADM or PTX for 48 hours, and drug sensitivity was confirmed with CCK8 assay. The OD values were measured at 450 nm using a microplate reader (Thermo, Schwerte, Germany). The IC_50_ for each drug was estimated using growth-inhibitory curves.

### Plate Colony Formation Assay

The 12-well plates were seeded with 300 cells per well and treated with different drugs. The drug concentrations for MCF-7/MDR, MCF-7, and MDA-MB-231 cells were ADM (MCF-7/MDR: 50 nM; MCF-7: 4 nM; MDA-MB-231: 25 nM) and PTX (MCF-7/MDR: 600 nM; MCF-7: 2 nM; MDA-MB-231: 45 nM). After 48 hours, the medium was replaced with fresh DMEM, and the solution was changed every 2 to 3 days until the colonies became visible to the naked eye. The colonies were washed and fixed, stained with 5% crystal violet, and then counted.

### Transmission Electron Microscope Observation of Autophagic Vesicles

Cells were fixed with glutaraldehyde and osmium acid before and after fixation, ethanol gradient dehydration, and Epon812 epoxy resin embedding, respectively, to prepare ultrathin sections. After double staining with bis-uranyl acetate and lead citrate, the autophagic vesicles with vacuolated bilayer-like structures were observed, as well as autophagic vesicles formed by double-layered membranes surrounding mitochondria and other organelles.

### Immunofluorescence (IF)

After treatment with ADM (MCF-7: 8 nM; MDA-MB-231: 50 nM) and PTX (MCF-7: 4 nM; MDA-MB-231: 90 nM) for 48 hours, cells were sequentially fixed with 4% paraformaldehyde, permeabilized with Triton-X 100, blocked with BSA, incubated with primary and secondary antibodies, and the nuclei were stained with DAPI (Meilune, China) before observation.

### Xenograft Model

All animal studies were approved by the Experimental Animal Ethical Committee of Jilin University. Female BALB/c-nu mice (4 weeks old, 16-20 g, specific pathogen-free standard) were purchased from Beijing Vital River Laboratory Animal Technology Co., Ltd., and were maintained in the Animal Experiment Center of the School of Basic Medicine, Jilin University (Laboratory Animal Use License No.SYXK (Jilin) 2023-0010).

5 × 10^6^ cells in 100 µL PBS were injected subcutaneously to establish the xenograft model. To clarify the role of CLDN6 in breast cancer chemoresistance, the following two groups were established: MDA-MB-231/Vec+ADM and MDA-MB-231/CLDN6+ADM. To further investigate whether CLDN6 induces breast cancer chemoresistance via protective autophagy, the following four groups were established: MDA-MB-231/CLDN6+control, MDA-MB-231/CLDN6+CQ, MDA-MB-231/CLDN6+ADM, and MDA-MB-231/CLDN6+CQ +ADM. Each group consisted of five nude mice. Administration of ADM was started 7 days after the injection of tumor cells, and ADM was administered via intraperitoneal injection at a dose of 5 mg/kg/3 days; CQ was administered by intraperitoneal injection at a dose of 30 mg/kg/3 days; MDA-MB-231/CLDN6+CQ +ADM group was injected with CQ for 8 hours followed by ADM injection; the MDA-MB-231/CLDN6+control group was given the same dose of saline solution. The mice were euthanised by CO_2_ asphyxiation after treatment. The length (L) and width (W) of tumor volume (V) were measured every three days. The tumor volume was estimated using the formula V = 0.5 × L × W^2^.

### Co-Immunoprecipitation (Co-IP) Assay

Cells were lysed with IP lysate buffer and the protein concentration was measured. Take 400 µg of protein, add IgG homologous to the target antibody, and 20 µL of Protein A/G Agarose Beads (Santa Cruz, CA, USA) to shake slowly for 30 minutes at 4 °C; centrifuge the precipitate at 2500 rpm at 4°C for 5 minutes, and then remove the non-specific binding proteins. Add the specific primary antibody to the total protein and incubate overnight at 4 °C. The protein-antibody complex was pulled down with Protein A/G Agarose Beads. After 4 hours, collect the beads and then boil them with 1×SDS-PAGE buffer, and the immunoprecipitated protein was eluted for WB analysis.

### Immunoelectron Microscopy Assay

After treatment of CQ (10 µM, 24 hours), cells were fixed in a mixture of paraformaldehyde and glutaraldehyde fixative, rinsed, dehydrated in ethanol, permeabilized with LR white acrylic resins (Sigma-Aldrich, St. Louis, MO, USA), embedded, and polymerized at 50-55 °C for 24 hours. Subsequently, the cells were blocked with BSA, and incubated with primary antibody and colloidal gold secondary antibody (Sigma-Aldrich, St. Louis, MO, USA). Finally, the cells were stained with dioxygen acetate and photographed.

### RNA Extraction and RT-PCR

Total RNA was isolated using the TRIzol reagent (Invitrogen, Carlsbad, CA, USA), and was reverse transcribed into cDNA using MonaScript RT All-in-One Mix with dsDNase (Monad, Wuhan, China). The PCR products were electrophoresed and subjected to autoradiography. Primers were synthesized by Sangon (Shanghai, China) and listed in Supplementary Table S2.

### Chromatin immunoprecipitation (ChIP) assay

ChIP assay was conducted following the manufacturer's protocol (Beyotime, Shanghai, China). DNA was extracted by DNA Purification Kit (Beyotime, Shanghai, China) and eluted for PCR. Primers were synthesized by Sangon (Shanghai, China) and listed in Supplementary Table S2.

### Statistical analysis

All statistical analyses were conducted using GraphPad Prism 8.0 (GraphPad, San Diego, CA, USA). The data were presented as the mean ± standard deviation (SD) at least three independent experiments. Significant differences were determined using Student's t-test and one-way ANOVA. *P* < 0.05 was considered statistically significant.

## Results

### CLDN6 is a potential biomarker for the chemosensitivity of breast cancer patients

**Figure 1:**
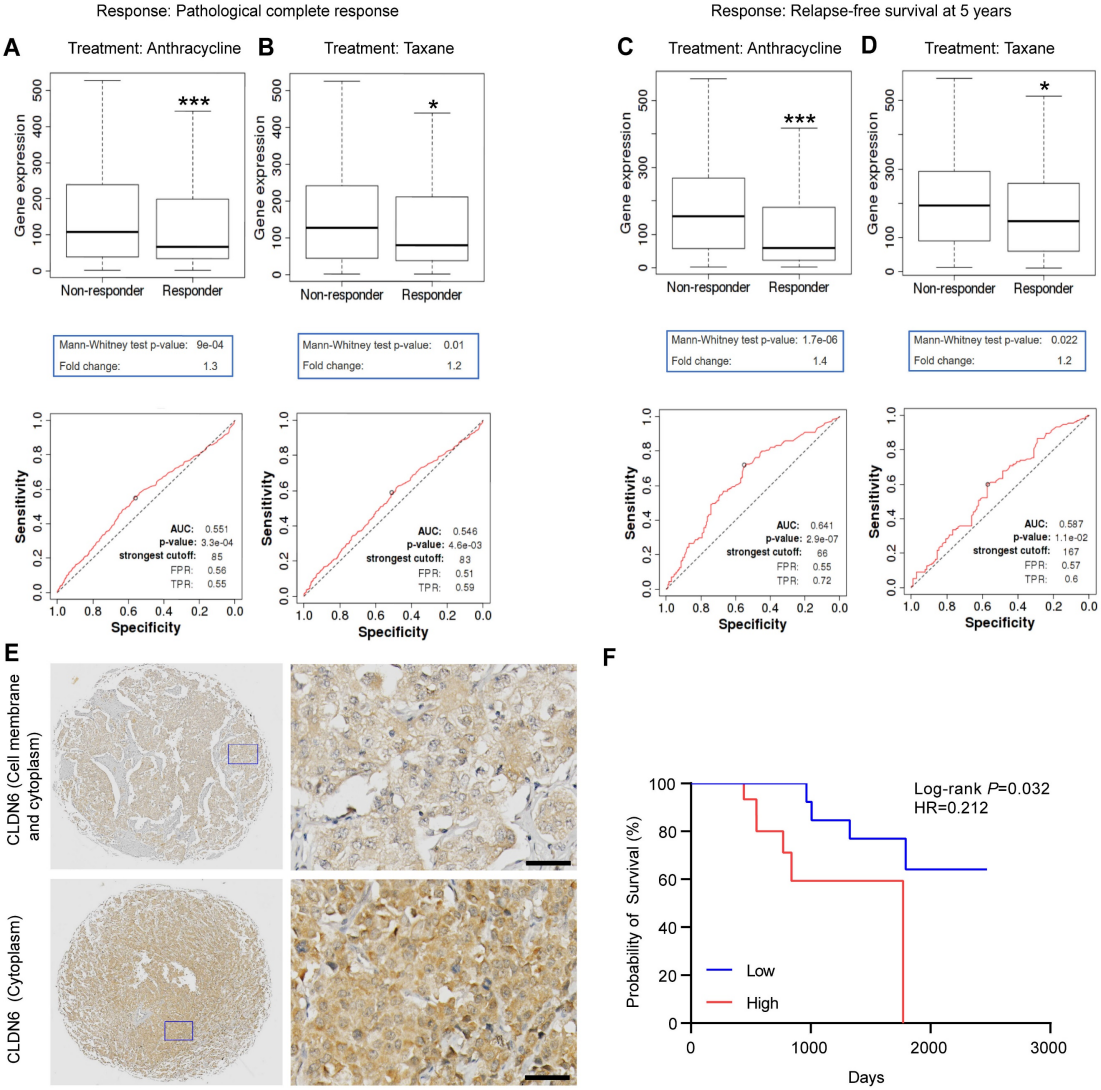
** CLDN6 is a potential biomarker of chemosensitivity in breast cancer patients.** The ROC Plotter online platform was used to analyze the expression of CLDN6 in patients who responded and did not respond to anthracyclines and taxane. Response: Pathological complete response (A-B) and relapse-free survival at 5 years (C-D); (E) Representative images of IHC staining of breast cancers in CLDN6 expression; (F) Kaplan-Meier survival curves for breast cancer patients as indexed by the H-score of CLDN6. Scale bar, 20 μm. *P < 0.05, ***P < 0.001.

First, we investigated the effect of CLDN6 expression on chemotherapy response and validated the prognostic value of CLDN6 expression on chemotherapy sensitivity using the ROC plotter. The results revealed that CLDN6 expression was significantly higher in the non-responder group compared to the responder group for anthracycline and taxane treatments. (Figure 1A-D). Based on ROC analysis, we identified CLDN6 as a potential biomarker for predicting the efficacy of anthracycline (Figure 1A and C) and taxane (Figure 1B and D). Furthermore, we performed IHC staining on 36 TNBC tissue samples from patients treated with chemotherapy to evaluate the relationship between CLDN6 expression and post-chemotherapy survival outcomes. The results showed that CLDN6 was predominantly localized in the cell membrane and cytoplasm (Figure 1E). Patients receiving chemotherapy were divided into two groups (CLDN6 high-expression and CLDN6 low-expression) based on the H-Score, and survival analysis revealed that patients with high CLDN6 expression experienced shorter overall survival (OS) after chemotherapy (Figure 1F). These findings suggest that CLDN6 may serve as a biomarker for chemosensitivity in breast cancer patients and hold significant clinical potential.

### CLDN6 promotes chemoresistance in breast cancer

**Figure 2:**
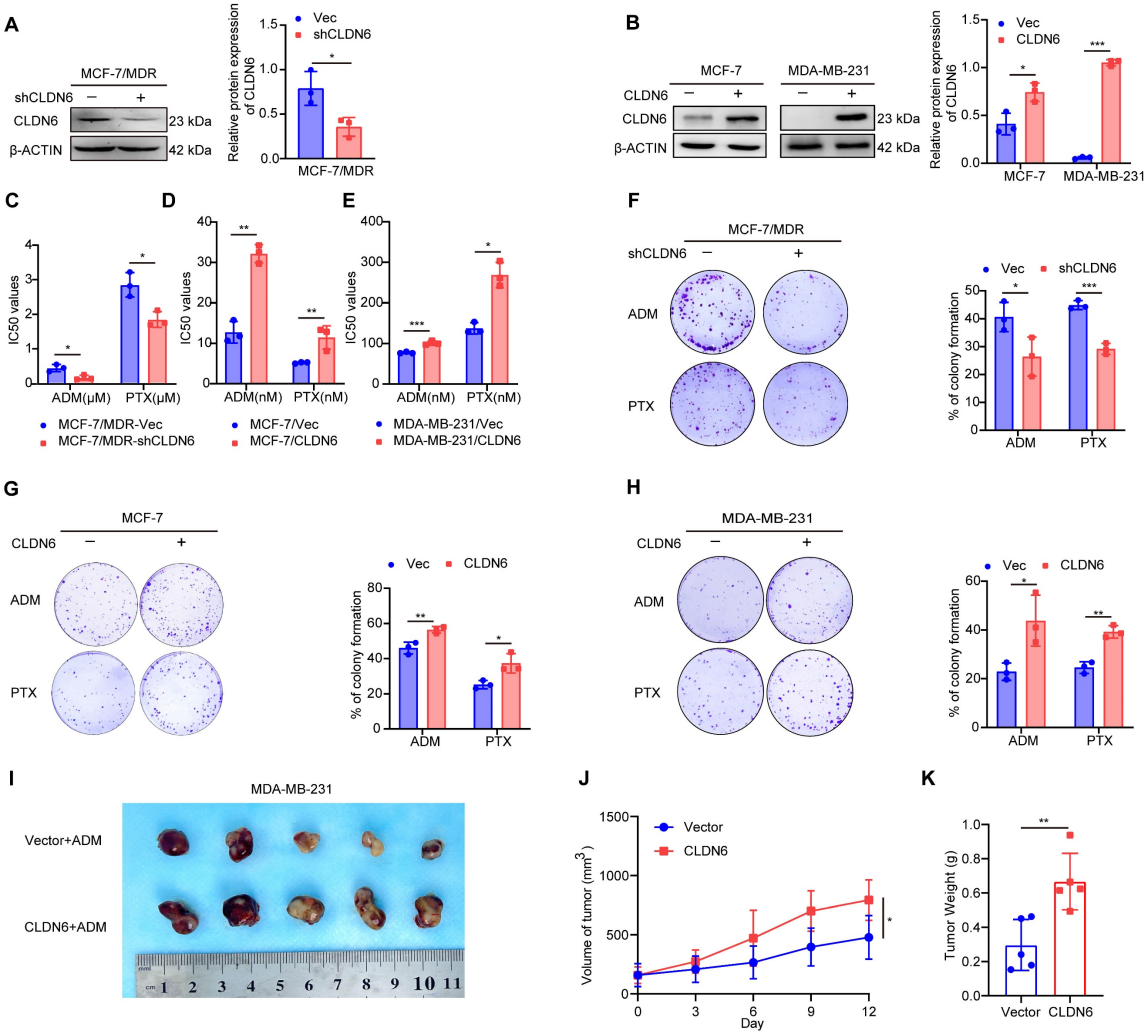
** CLDN6 promotes chemoresistance in breast cancer.** (A-B) WB was performed to examine the knockdown and overexpression efficiency of CLDN6; (C-E) IC_50_ values of MCF-7/MDR cells silencing CLDN6, MCF-7 and MDA-MB-231 cells overexpressing CLDN6 on ADM and PTX; (F) Plate colony formation assay to detect the effect of silencing CLDN6 on the clone formation ability of MCF-7/MDR cells after treated with ADM (50 nM) and PTX (600 nM); (G-H) Plate colony formation assay to detect the effect of overexpressing CLDN6 on the clone formation ability of MCF-7 and MDA-MB-231 cells in response to ADM (MCF-7: 4 nM; MDA-MB-231: 25 nM) and PTX (MCF-7: 2 nM; MDA-MB-231: 45 nM); (I) Images of subcutaneous xenograft cancers (5 per group). (J-K) Tumor weight and volume were measured. *P < 0.05, **P < 0.01, ***P < 0.001.

Next, we investigated the role of CLDN6 in chemoresistance at the cellular level. Our previous studies have demonstrated that CLDN6 is highly expressed in MCF-7/MDR cells and lowly expressed in MCF-7 and MDA-MB-231 cells 11, 23. Therefore, we knocked down CLDN6 in MCF-7/MDR cells (Figure 2A) and overexpressed CLDN6 in MCF-7 and MDA-MB-231 cells (Figure 2B) separately. The above cells were treated with ADM and PTX, followed by an assessment of cell viability and colony formation ability. The CCK8 results revealed that CLDN6 knockdown markedly reduced the IC_50_ values in MCF-7/MDR cells (Figure 2C), while CLDN6 overexpression significantly increased the IC_50_ values in MCF-7 and MDA-MB-231 cells (Figure 2D-E). Simultaneously, the plate colony formation results demonstrated a marked reduction in colony number in the MCF-7/MDR-shCLDN6 group (Figure 2F), and a notable increase in the MCF-7/CLDN6 and MDA-MB-231/CLDN6 groups (Figure 2G-H). To corroborate our *in vitro* findings, we established xenograft implantation tumor models. As shown in Figure 2I-K, compared with the MDA-MB-231/Vec+ADM group, the tumor volume and weight were higher in the MDA-MB-231/CLDN6+ADM group. Collectively, these results suggest that CLDN6 promotes chemoresistance in breast cancer.

### CLDN6 promotes autophagy in response to ADM and PTX

**Figure 3:**
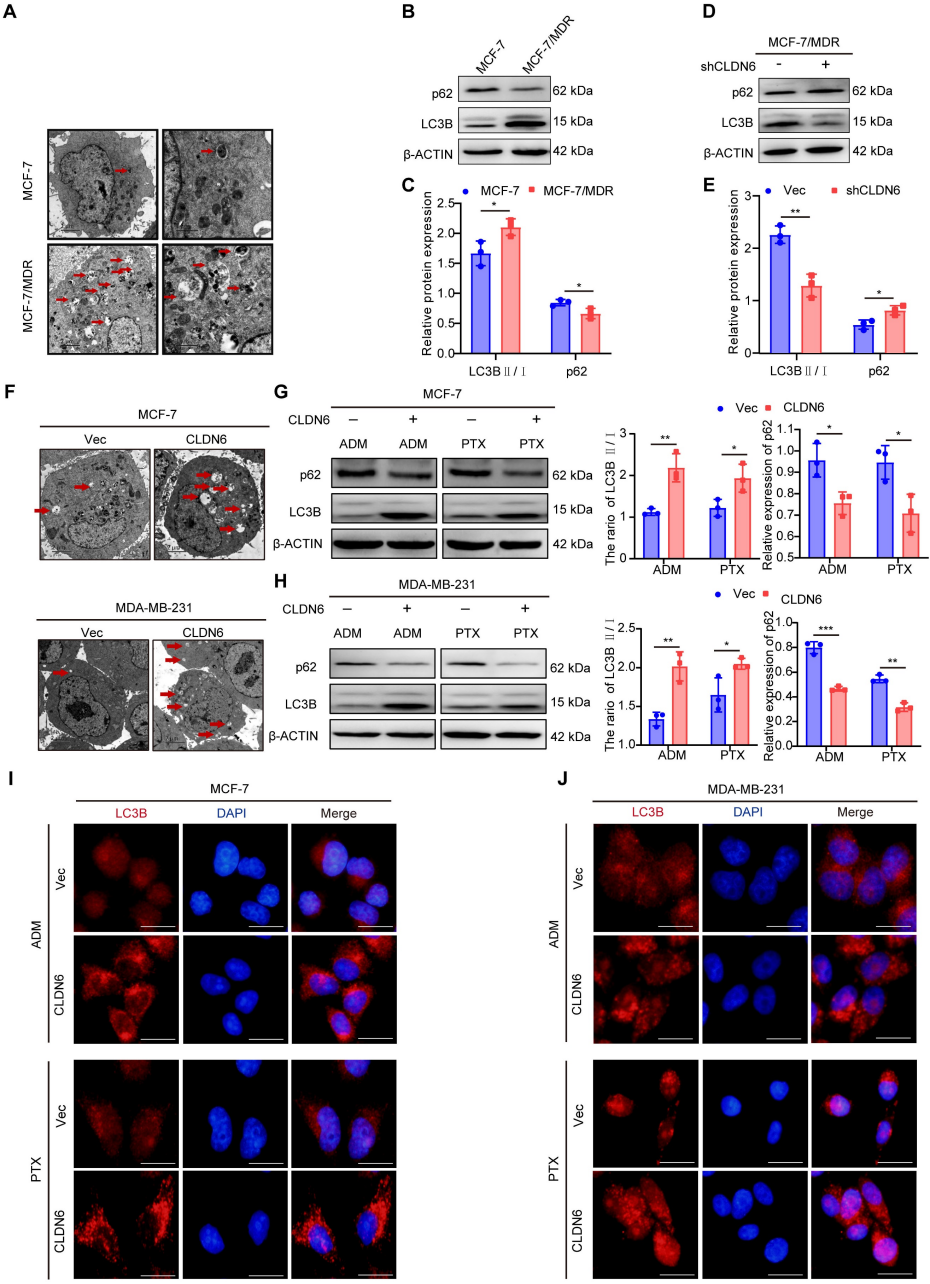
** CLDN6 promotes autophagy in response to ADM and PTX.** (A) Transmission electron microscopy observation of autophagosomes in MCF-7 and MCF-7/MDR cells. (B-C) WB to detect the expression of LC3B Ⅱ/Ⅰ and p62 in MCF-7 and MCF-7/MDR cells. (D-E) WB to detect the expression of LC3B Ⅱ/Ⅰ and p62 in MCF-7/MDR-shCLDN6 cells. (F) Transmission electron microscopy observation of autophagosomes in MCF-7/CLDN6 and MDA-MB-231/CLDN6 cells in response to ADM (MCF-7: 8 nM; MDA-MB-231: 50 nM). Red arrows, autophagosomes; (G-H) WB analysis of LC3B Ⅱ/Ⅰ and p62 expression in MCF-7/CLDN6 and MDA-MB-231/CLDN6 cells in response to ADM (MCF-7: 8 nM; MDA-MB-231: 50 nM) and PTX (MCF-7: 4 nM; MDA-MB-231: 90 nM); (I-J) IF assay to detect the effect of overexpression of CLDN6 on the number of LC3B puncta in MCF-7 and MDA-MB-231 cells under ADM and PTX treatment conditions. (n=3). Scale bar, 50 μm. *P < 0.05, **P < 0.01, ***P < 0.001.

Numerous studies have confirmed that autophagy can promote chemoresistance 2, 24. Transmission electron microscopy analysis revealed a greater number of autophagosomes in MCF-7/MDR compared with MCF-7 cells (Figure 3A). Furthermore, the WB results showed that the LC3B II/I ratio was increased and p62 expression was decreased significantly in MCF-7/MDR cells (Figure 3B-C). These results suggest that MCF-7/MDR cells exhibit a higher level of autophagy compared with MCF-7 cells. To determine whether the elevated level of autophagy in MCF-7/MDR cells is associated with CLDN6, we detected the effect of silencing CLDN6 on autophagy levels in MCF-7/MDR cells. The WB results showed a decrease in the LC3B-II/I ratio and an increase in p62 expression (Figure 3D-E). These data point out that CLDN6 promotes autophagy in MCF-7/MDR cells. To further elucidate the role of CLDN6 in promoting autophagy under chemotherapy, we examined the level of autophagy in MCF-7/CLDN6 and MDA-MB-231/CLDN6 cells following treatment with ADM and PTX. The transmission electron microscopy analysis revealed that cells overexpressing CLDN6 contained a higher number of autophagosomes compared to the vector group. (Figure 3F). Additionally, the WB results showed that the ratio of LC3B II/I increased and p62 expression decreased in MCF-7/CLDN6 and MDA-MB-231/CLDN6 cells (Figure 3G-H). Besides, the IF results showed that the number of LC3B puncta following CQ treatment was higher in the CLDN6 overexpression group compared to the vector group. (Figure 3I-J). The above results indicate that CLDN6 is involved in chemotherapy-induced autophagy.

### CLDN6 induces breast cancer chemoresistance via protective autophagy

**Figure 4:**
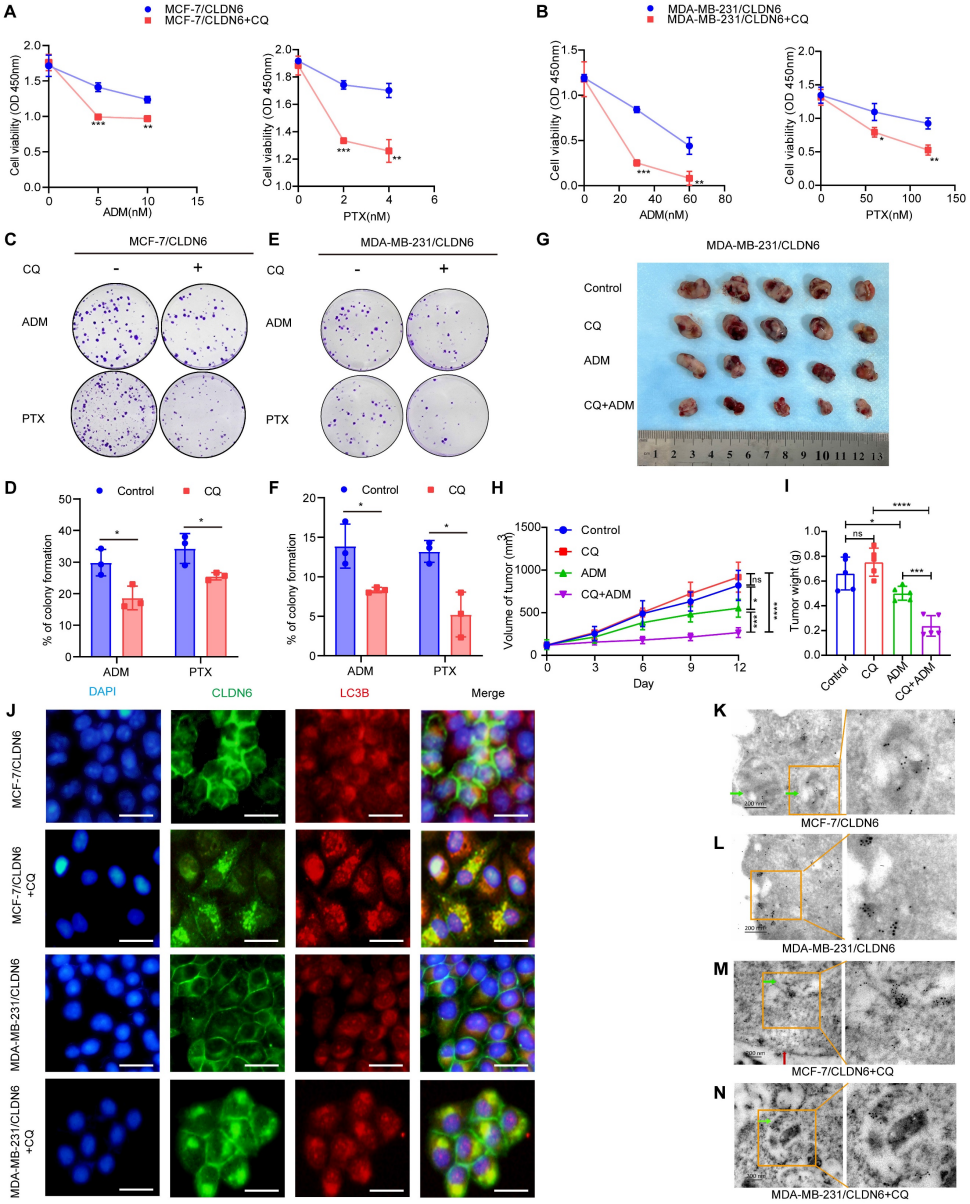
** CLDN6 induces breast cancer chemoresistance via protective autophagy.** (A-B) CCK8 assay to detect the effect of CQ on MCF-7/CLDN6 and MDA-MB-231/CLDN6 cell viability. (C-F) Cells were treated with CQ (10 µM, 24h) followed by ADM and PTX to detect the effect of CQ on the number of colonies of MCF-7/CLDN6 and MDA-MB-231/CLDN6 cells. (G) Images of subcutaneous xenograft cancers (5 per group). (H-I) Tumor weight and volume were measured. (J) IF assay to observe the co-localization of CLDN6 and LC3B after CQ treatment. Scale bar, 50 μm. (K-N) Immunoelectron microscopy to detect the subcellular localization of CLDN6 and LC3B before and after CQ treatment. Colloidal gold (LC3B: 5 nm; CLDN6: 10 nm). Green arrows, autophagosomes; red arrows, cell membrane. Scale bar, 200 nm. *P < 0.05, **P < 0.01, ***P < 0.001, ****P < 0.0001, ns, no significance.

To investigate whether CLDN6 promotes chemoresistance in breast cancer cells through protective autophagy, we treated MCF-7/CLDN6 and MDA-MB-231/CLDN6 cells with the autophagy inhibitor CQ. The CCK8 and colony formation assay revealed that under ADM and PTX treatment, CQ significantly inhibited cell viability (Figure 4A-B) and the number of colonies (Figure 4C-F) in MCF-7/CLDN6 and MDA-MB-231/CLDN6 cells. To corroborate our *in vitro* findings, we established xenograft implantation tumor models. As shown in Figure 4G-I, tumor volume and weight were significantly reduced in the MDA-MB-231/CLDN6+ADM group compared to the MDA-MB-231/CLDN6+control group, whereas no significant changes were observed in the MDA-MB-231/CLDN6+CQ group. Tumor volume and weight in the MDA-MB-231/CLDN6+CQ+ADM group were significantly smaller than those in the MDA-MB-231/CLDN6+ADM or MDA-MB-231/CLDN6+CQ group. Taken together, these findings suggest that CLDN6 promotes chemoresistance in breast cancer cells through the induction of protective autophagy both *in vitro* and *in vivo*.

Interestingly, when cells were treated with CQ, we observed an unexpected increase in the punctate distribution of CLDN6 in the cytoplasm of MDA-MB-231/CLDN6 cells (Figure S1A). Furthermore, the IF results showed that CLDN6 and LC3B co-localized in the cytoplasm (Figure 4J). The Co-IP results showed that CLDN6 and LC3B interacted with each other (Figure S1B). To further investigate the ultrastructural localization and interaction of CLDN6 and LC3B, we conducted immunoelectron microscopy experiments using MCF-7/CLDN6 and MDA-MB-231/CLDN6 cells. The results showed that, in the absence of CQ treatment, LC3B was localized within autophagosomes (Figure 4K), and CLDN6 co-localized with LC3B in the cytoplasm adjacent to the cell membrane (Figure 4L). Following CQ treatment, both proteins were found to co-localize near the autophagosome membranes (Figure 4M-N). These findings demonstrate that CLDN6 co-localizes with LC3B in autophagosomes in response to CQ. Subsequently, we treated MCF-7/CLDN6 and MDA-MB-231/CLDN6cells with 3-Methyladenine (3-MA), Bafilomycin A1 (Baf-A1), and CQ, and the WB results showed that CLDN6 expression increased after autophagy inhibition (Figure S1C). Collectively, these results suggest that CLDN6 may undergo degradation through the autophagic pathway via binding to LC3B, indicating that autophagy has a negative feedback regulatory effect on CLDN6 expression.

### CLDN6 mediates protective autophagy via AMPK/ULK1 signaling

**Figure 5:**
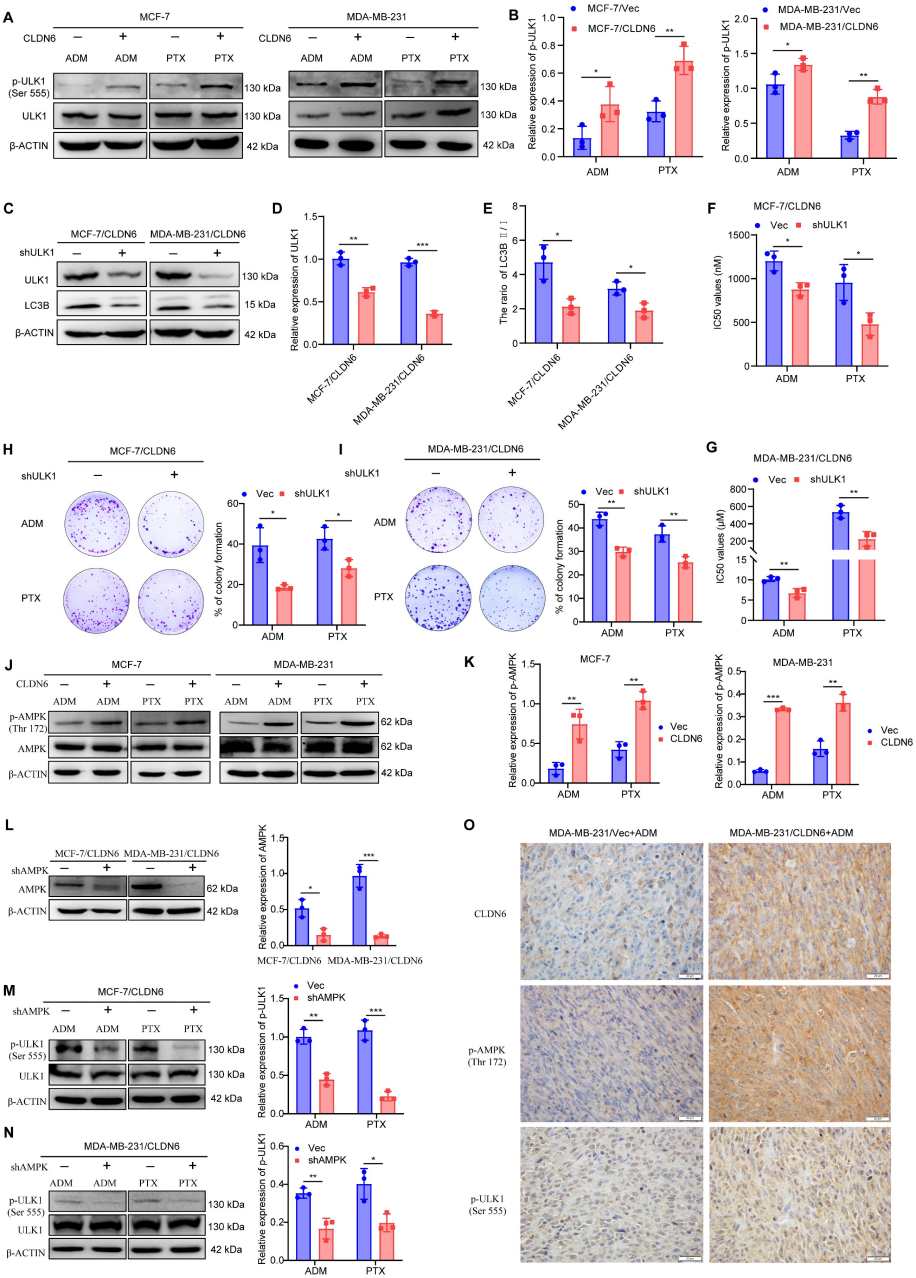
** CLDN6 mediates protective autophagy via AMPK/ULK1 signaling.** (A-B) WB was used to detect the effect of CLDN6 on the expression of p-ULK1 in response to ADM and PTX. (C-E) WB to verify the silencing effect of ULK1 and to detect the expression of LC3B. (F-G) IC_50_ values of MCF-7/CLDN6 and MDA-MB-231/CLDN6 cells silencing ULK on ADM and PTX. (H-I) Plate colony formation assay to detect the effect of silencing ULK1 on the number of colonies of MCF-7/CLDN6 and MDA-MB-231/CLDN6 cells in response to ADM (MCF-7/CLDN6: 300 nM; MDA-MB-231/CLDN6: 2 µM) and PTX (MCF-7/CLDN6: 160 nM; MDA-MB-231/CLDN6: 80 µM); (J-K) WB to detect the effect of CLDN6 on the expression of p-AMPK. (L) WB to verify the silencing effect of AMPK. (M-N) WB to detect the effect of silencing AMPK on the expression of p-ULK1 in breast cancer cells overexpressing CLDN6. (O) Representative images of CLDN6, p-AMPK, and P-ULK1 expression in transplanted tumor tissues. (n=3). Scale bar, 20 μm. *P < 0.05, **P < 0.01, ***P < 0.001.

Our previous mRNA sequencing of MCF-7/CLDN6 cells showed significant alterations in the expression of 15 autophagy-related genes, including ULK1 (Figure S2). ULK1 is a crucial protein involved in the initiation of autophagy and is activated through phosphorylation 25. Therefore, we investigated the effect of CLDN6 on ULK1 activation under chemotherapeutic conditions. The WB results showed that the expression of p-ULK1 notably increased in MCF-7/CLDN6 and MDA-MB-231/CLDN6 cells (Figure 5A-B). Subsequently, we knocked down ULK1 in MCF-7/CLDN6 and MDA-MB-231/CLDN6 cells (Figure 5C-D), and examined the effects on autophagy and chemoresistance under ADM and PTX. The WB results showed that ULK1 knockdown significantly decreased the ratio of LC3B II/I (Figure 5C and E). CCK8 results showed that ULK1 knockdown led to a decrease in the IC_50_ values (Figure 5F-G). The plate colony formation assay showed that ULK1 knockdown prominently inhibited the clonogenic ability of MCF-7/CLDN6 and MDA-MB-231/CLDN6 cells (Figure 5H-I). These results indicate that CLDN6 relies on ULK1 to induce protective autophagy.

Given that ULK1 activation is regulated by AMPK 26, we next examined the impact of CLDN6 on AMPK activation in response to ADM and PTX. The WB results showed that the expression of p-AMPK significantly increased in MCF-7/CLDN6 and MDA-MB-231/CLDN6 cells (Figure 5J-K). Next, AMPK was silenced in breast cancer cells overexpressing CLDN6 (Figure 5L), and the WB results showed a notable decrease in the expression of p-ULK1 (Figure 5M-N). Furthermore, we performed IHC staining of CLDN6, p-AMPK, and p-ULK1 in xenograft tumor tissues from MDA-MB-231/Vec+ADM and MDA-MB-231/CLDN6+ADM groups, and the results showed that p-AMPK and p-ULK1 were predominantly localized in the cytoplasm, and their expression was higher in MDA-MB-231/CLDN6+ADM group than that in MDA-MB-231/Vector+ADM group (Figure 5O). The results suggest that CLDN6 induces protective autophagy through AMPK/ULK1 signaling.

### CLDN6 interacts with LKB1 through its PDZ-binding motif and activates AMPK/ULK1 signaling

**Figure 6:**
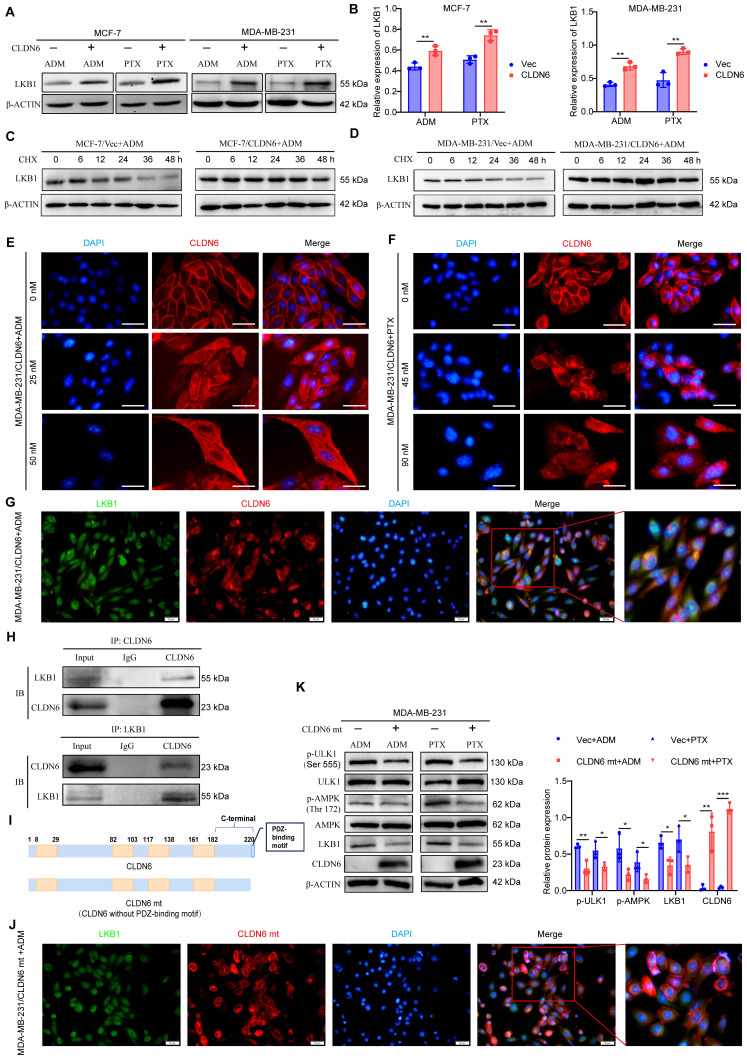
** CLDN6 interacts with LKB1 through its PDZ-binding motif and activates AMPK/ULK1 signaling.** (A-B) WB was performed to detect the effect of CLDN6 on the expression of LKB1; (C-D) WB to detect the effect of CLDN6 on the degradation of LKB1 in response to ADM after CHX (10 µM) treatment. (E-F) IF to detect the effect of ADM and PTX on the subcellular localization of CLDN6. Scale bar, 50 μm. (G) IF to detect the co-localization of CLDN6 and LKB1 in response to ADM in MDA-MB-231/CLDN6 cells. Scale bar, 50 μm. (H) Co-IP was performed to detect the interaction between LKB1 and CLDN6 in MDA-MB-231/CLDN6 cells in response to ADM. (I) Schematic structure of CLDN6 and CLDN6 mt; (J) IF to detect the co-localization of CLDN6 and LKB1 in response to ADM in MDA-MB-231/CLDN6mt cells. (K) WB to detect the effect of CLDN6 mt on the expression of LKB1, p-AMPK, and p-ULK1. (n=3). Scale bar, 50 μm. *P < 0.05, **P < 0.01, ***P < 0.001.

LKB1 is a crucial upstream regulator for AMPK signaling, so we examined the effect of CLDN6 overexpression on LKB1 expression in MCF-7 and MDA-MB-231 cells under ADM and PTX stimulation. The RT-PCR results showed that LKB1 mRNA expression was not significantly altered (Figure S3A-B). In contrast, the WB results revealed that LKB1 protein expression was notably increased in MCF-7/CLDN6 and MDA-MB-231/CLDN6 cells (Figure 6A-B). Additionally, we used CHX to inhibit protein synthesis, and the WB results showed that the LKB1 degradation rate was lower in the MCF-7/CLDN6 and MDA-MB-231/CLDN6 groups compared to the vector group (Figure 6C-D). The above data demonstrate that CLDN6 inhibits the degradation of LKB1.

We next explored the potential mechanisms by which CLDN6 regulates LKB1. First, we unexpectedly found that cytoplasmic localization of CLDN6 was increased in MCF-7/CLDN6 and MDA-MB-231/CLDN6 cells following treatment with ADM and PTX (Figure 6E-F). Given that LKB1 is widely distributed in the cytoplasm, we next examined the colocalization of CLDN6 and LKB1 under ADM treatment using IF assay. The results demonstrated a clear co-localization of CLDN6 and LKB1 in the cytoplasm (Figure 6G). Furthermore, the interaction between CLDN6 and LKB1 was confirmed using CO-IP assay (Figure 6H). The PDZ-binding motif of CLDN6 is a critical structure for mediating protein-protein interactions. To investigate its functional role, we generated a CLDN6 mutant (CLDN6 mt) with the PDZ-binding motif deleted and overexpressed CLDN6 mt in MCF-7 and MDA-MB-231 cells (Figure 6I and S4A). Notably, CLDN6 mt was predominantly localized in the cell membrane and was virtually absent in the cytoplasm (Figure S4B-C). Following ADM treatment, CLDN6 mt was predominantly located in the cell membrane and did not significantly co-localize with LKB1 in the cytoplasm in MDA-MB-231/CLDN6 mt cells (Figure 6J). CLDN6 mt inhibited the expression of LKB1, the activation of p-AMPK and p-ULK (Figure 6K). These findings suggest that CLDN6 interacts with LKB1 through its PDZ-binding motif and activates AMPK/ULK1 signaling in response to ADM and PTX.

### Chemotherapy promotes CLDN6 expression via the ROS/GATA4 axis

**Figure 7:**
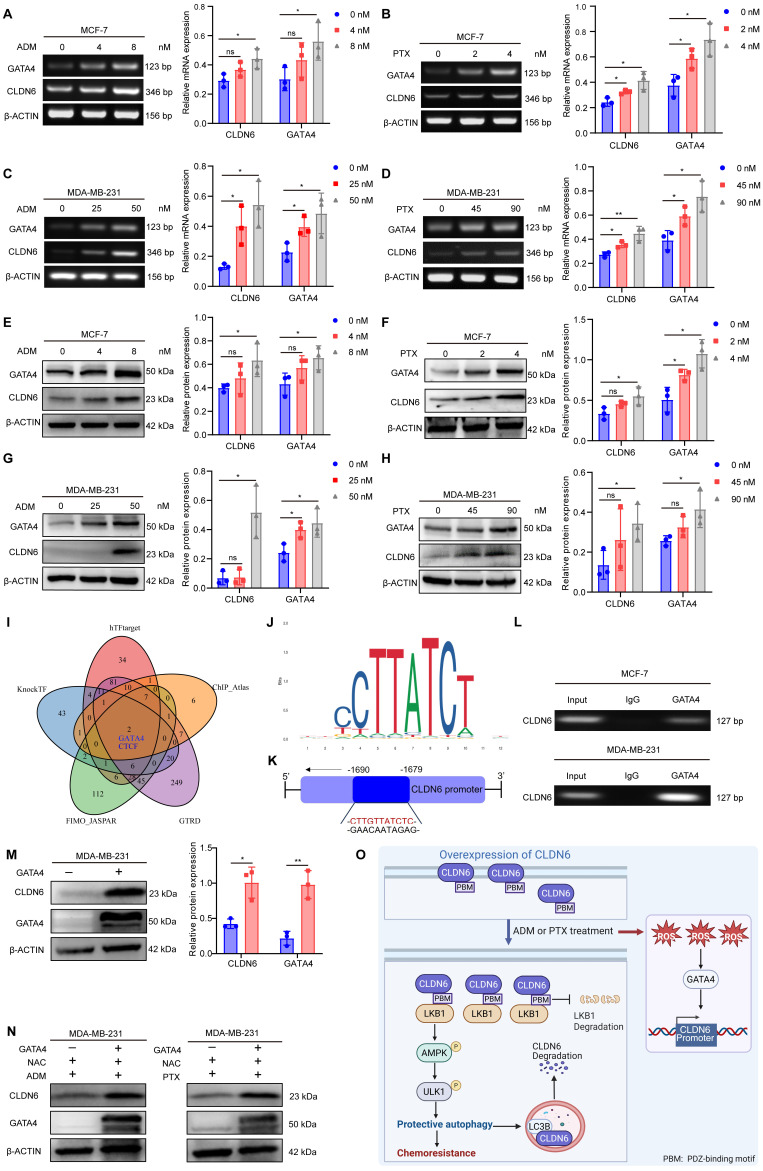
** Chemotherapy promotes CLDN6 expression via the ROS/GATA4 axis.** (A-D) RT-PCR to detect the effects of ADM and PTX on mRNA expression of CLDN6 and GATA4 in MCF-7 and MDA-MB-231 cells. (E-H) WB to detect the effects of ADM and PTX on protein expression of CLDN6 and GATA4 in MCF-7 and MDA-MB-231 cells. (I) hTFtarget, KnockTF, FIMO_JASGTRD, GTRD, and ChIP_Atlas to predict the transcription factors of CLDN6. (J-K) JASPAR database analysis of GATA4 binding sites in the CLDN6 promoter region and schematic diagrams. (L) ChIP to detect the binding of GATA4 to CLDN6 promoter in MCF-7 and MDA-MB-231 cells. (M) WB to detect the GATA4 overexpression efficiency and the effect on CLDN6 expression. (N) WB to detect the effect of GATA4 overexpression on CLDN6 expression under NAC treatment (5 mM). (O) A suggested framework elucidating the regulatory process by which CLDN6 promotes chemoresistance of breast cancer via protective autophagy. (n=3). *P < 0.05, **P < 0.01, ns, no significance.

The promotion of pro-survival protein expression by chemotherapy is one of the mechanisms underlying chemotherapy resistance 27. Therefore, we investigated the impact of chemotherapy on the expression of CLDN6 in MCF-7 and MDA-MB-231 cells. The RT-PCR and WB results demonstrated that ADM and PTX induced a dose-dependent increase in both CLDN6 mRNA (Figure 7A-D) and protein (Figure 7E-H) expression. Next, we utilized hTFtarget, knockTF, FIMO_JASPAR, GTRD, and ChIP_Atlas databases to identify potential transcription factors for CLDN6 through intersection analysis. The results indicated that GATA4 is one of the potential transcription factors regulating CLDN6 expression. (Figure 7I). The RT-PCR and WB results showed that ADM and PTX promoted the mRNA (Figure 7A-D) and protein expression (Figure 7E-H) of GATA4. JASPAR database (https://jaspar.elixir.no/) analysis demonstrated the presence of a highly probable binding site for GATA4 within the promoter region of CLDN6 (Figure 7J). Consequently, the ChIP assay was conducted to validate the interplay of GATA4 and the promoter region of CLDN6 in MCF-7 and MDA-MB-231 cells under ADM treatment. The results showed that GATA4 bound to the CLDN6 promoter region at the CTTGTTATCTC sequence (Figure 7K-L). It is known that chemotherapy can induce the production of ROS, and the transcription of GATA4 is positively regulated by ROS 22. To further illustrate the role of the ROS/GATA4 axis in chemotherapy-induced CLDN6 expression, we treated MCF-7 and MDA-MB-231cells with the ROS inhibitor NAC. We observed a significant reduction in the expression of both GATA4 and CLDN6 (Figure S5A-C). Next, we overexpressed GATA4 in MDA-MB-231 cells, and the WB results indicated a significant increase in CLDN6 expression in the GATA4 overexpression group (Figure 7M). Furthermore, we treated MDA-MB-231/GATA4 cells with NAC in combination with either ADM or PTX, and then detected the expression of CLDN6. The WB results showed that CLDN6 expression was notably elevated in MDA-MB-231/GATA4 cells (Figure 7N and S5D-E). This suggests that in MCF-7 and MDA-MB-231 cells, ADM promotes the expression of GATA4 by inducing ROS production, which in turn promotes CLDN6 expression.

## Discussion

Beyond its traditional barrier function, CLDN6 also participates in signaling through its PDZ-binding motif 7, 10, playing a pivotal role in the growth, metastasis, and chemoresistance of various tumors. Consequently, it emerges as a promising target for cancer therapy 6, 28. CLDN6 promotes or inhibits breast cancer depending on its subcellular localization and different stress conditions. Our latest study revealed that CLDN6 is mainly located in the cell membrane and inhibits the growth and metastasis of breast cancer. 29. In the present study, we demonstrated that CLDN6 promotes breast cancer growth under ADM and PTX treatment through both *in vitro* and *in vivo* experiments, indicating that CLDN6 induces breast cancer chemoresistance. Based on ROC plotter and TMA, we identified that CLDN6 is a potential biomarker for the chemoresistance of breast cancer patients. Through further mechanistic studies, we found that in MCF-7/CLDN6 and MDA-MB-231/CLDN6 cells, cytoplasmic CLDN6 binds to LKB1 through its PDZ-binding motif, leading to the activation of AMPK/ULK1 signaling and the promotion of protective autophagy following ADM and PTX treatment. This suggests that under chemotherapy conditions, cytoplasmic CLDN6 induces breast cancer chemoresistance through protective autophagy.

Protective autophagy, a self-defense mechanism employed by cancer cells in response to chemotherapeutic stress, has been established as a critical contributor to cancer chemoresistance 30, 31. Cai et al. 32 reported that LncRNA GBCDRlnc1 promotes protective autophagy, contributing to the chemoresistance of gallbladder cancer. Chen et al. 33 found that SHMT2 reduces the stability of cytosolic p53 to induce autophagy, which maintains the survival of cancer cells treated with 5-FU. In gastric cancer cells, lncRNA EIF3J-DT is involved in the regulation of autophagy and chemoresistance 34. Notably, in addition to protective autophagy, non-protective autophagy may also occur in cells, and the two can only be distinguished by changes in cellular phenotype following autophagy inhibition 35. In this study, we utilized autophagy inhibitor CQ to treat MCF-7/CLDN6 and MDA-MB-231/CLDN6 cells, and found that cell viability and clone formation ability were significantly decreased after treatment of ADM and PTX. *In vivo* experiments confirmed that the volume and weight of transplanted tumors in the MDA-MB-231/CLDN6+CQ+ADM group were significantly smaller than those in the MDA-MB-231/CLDN6+ADM group. Our findings demonstrated that CLDN6 induced protective autophagy, which contributed to the resistance of breast cancer cells to chemotherapy *in vitro* and *in vivo*.

ULK1, a key protein for autophagy initiation, plays an important role in chemotherapy-induced protective autophagy. For instance, SNHG6 promotes chemoresistance by ULK1-mediated autophagy 36. LncRNA LUCAT1 contributes to the resistance of NSCLC cells to cisplatin by regulating the miR-514a-3p/ULK1 axis 37. Currently, there are limited studies on CLDNs and ULK1. It was only found that CLDN1 promotes tumor cell proliferation, metastasis, and protective autophagy-mediated chemoresistance by activating ULK1 38, 39. In this study, we found that in MCF-7 and MDA-MB-231 cells, CLDN6 overexpression increased p-ULK1 expression and induced protective autophagy response to ADM and PTX. Of note, we have confirmed that CLDN6 inhibits breast cancer metastasis through actin cytoskeleton-mediated autophagy 10. Under the influence of estrogen, CLDN6 inhibits breast cancer metastasis by upregulating beclin1 to mediate autophagy 9. Therefore, it is believed that under different conditions, CLDN6 selectively activates specific signaling pathways, and regulates different autophagy-related proteins to induce different types of autophagy.

LKB1/AMPK signaling is the classic upstream regulator of ULK1 and is closely related to protective autophagy 18, 19, 40. Our data demonstrated that the protein level of LKB1 and p-AMPK expression was increased in MCF-7/CLDN6 and MDA-MB-231/CLDN6 cells. Importantly, we found that in MDA-MB-231/CLDN6 cells, CLDN6 interacted with LKB1 via its PDZ-binding motif in the cytoplasm following ADM treatment. In addition, we observed that CLDN6 inhibits the degradation of LKB1 protein under ADM treatment in MCF-7/CLDN6 and MDA-MB-231/CLDN6 cells. Therefore, we hypothesized that CLDN6 may prevent LKB1 degradation upon binding, although the specific mechanism remains to be further studied. After overexpression of the PDZ-binding motif-deleted mutant in MDA-MB-231 cells, CLDN6 was found to be mainly localized to the cell membrane, but rarely in the cytoplasm under ADM treatment. As a result, the expression of LKB1 and the activation of the AMPK/ULK1 signaling pathway were significantly inhibited. This indicates that, under chemotherapy conditions, the roles of CLDN6 in the cell membrane and cytoplasm are not the same. Similarly, different subcellular localizations of CLDN1 differentially regulate melanoma metastasis 41. However, the mechanism of PDZ binding motif-dependent translocation of CLDN6 from the cell membrane to the cytoplasm under chemotherapeutic conditions requires further exploration.

Stimuli such as hypoxia and starvation can alter the expression of CLDNs 8, 42. Our findings indicate that ADM and PTX treatment induced CLDN6 expression. Chemotherapeutic agents usually cause an increase in intracellular ROS levels, which serve as important signaling molecules that activate multiple signaling pathways within cells 43. In the present study, we found that ADM and PTX induced the expression of GATA4 by promoting ROS production in MCF-7 and MDA-MB-231 cells, which acts as a transcription factor for CLDN6. Further research is needed to determine whether targeting CLDN6 or the ROS/GATA4 axis could serve as a potential strategy for improving the efficacy of chemotherapy. Additionally, it would be valuable to investigate whether other stimuli, such as hypoxia and starvation, also affect the ROS/GATA4/CLDN6 axis and whether there are variations in the response across various cancer cell types.

Studies have found that negative feedback regulation of signaling pathways occurs in tumors in response to chemotherapy stimulation 44. Although autophagy has traditionally been regarded as a non-specific degradation process, recent studies have shown that it is also essential for the degradation of specific cargoes such as organelles and proteins 45, 46. Our observations revealed the colocalization of CLDN6 with LC3B in the cytoplasm following CQ treatment in MCF-7/CLDN6 and MDA-MB-231/CLDN6 cells, with the autophagy inhibitors (3-MA, Baf-A1, and CQ) seemingly enhancing CLDN6 protein expression. This suggests that CLDN6 is degraded via the autophagic pathway, which is consistent with a recent study indicating that AP2M1 mediates autophagy-induced degradation of CLDN2 through endocytosis and interaction with LC3B 47. However, further investigations are necessary to explore how CLDN6 enters the cytoplasm and co-localizes with LC3B.

## Conclusions

In summary, we found that CLDN6 promotes chemoresistance through the activation of protective autophagy in breast cancer. Mechanistically, CLDN6 interacted with LKB1 through its PDZ-binding motif, leading to the activation of AMPK/ULK1 signaling. This activation subsequently induces protective autophagy. Notably, chemotherapy enhanced CLDN6 expression through the ROS/GATA4 axis. Furthermore, autophagy exerted a negative feedback regulation on CLDN6 expression. Figure 7O shows this complete regulatory network. Our study presents a novel biomarker for chemotherapy-induced protective autophagy and establishes a theoretical and experimental foundation for targeting CLDN6 to enhance chemosensitivity in breast cancer patients.

## Supplementary Material

Supplementary figures and tables.

## Abbreviations

CLDN6: claudin-6
ADM: adriamycin
PTX: paclitaxel
ROS: reactive oxygen species
ULK1: unc-51 like autophagy activating kinase 1
ATGs: autophagy-related proteins
LKB1: liver kinase B1
IHC: immunohistochemistry
TMA: tissue microarray
TNBC: triple-negative breast cancer
pCR: pathological complete response
RFS: relapse-free survival
AUC: area under the curve
HEK: human embryonic kidney
FBS: fetal bovine serum
WB: western blot
CCK8: cell counting kit 8
CQ: chloroquine
IF: immunofluorescence
Co-IP: co-immunoprecipitation
ChIP: chromatin immunoprecipitation
OS: overall survival
3-MA: 3-methyladenine
Baf-A1: bafilomycin A1
