# Supplementary material for: CLDN6 induces chemoresistance through protective autophagy in breast cancer
Source: Int J Biol Sci. 2025 Aug 22;21(12):5444–59. doi: 10.7150/ijbs.116340 (PMC12435484; doi:10.7150/ijbs.116340)
Supplement: Supplementary file 1 — Supplementary figures and tables. [file ijbsv21p5444s1.pdf]

Figure S1

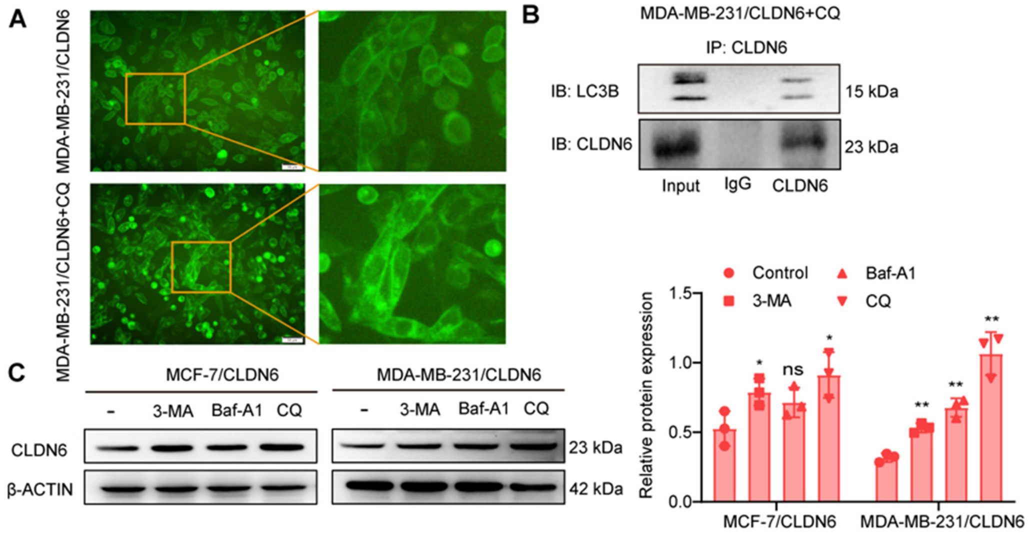

Figure S1. Effects of CQ on CLDN6 localization, interaction with LC3B, and expression

(A) The subcellular localization of CLDN6 before and after CQ action using fluorescence microscopy; (B) Co-IP to detect the interaction between CLDN6 and LC3B after CQ action; (C) Effect of autophagy inhibitors on the CLDN6 protein level in cells overexpressing CLDN6. (n=3). Scale bar, 50 μm. *$P < 0.05$, **$P < 0.01$, ns, no significance.

Figure S2

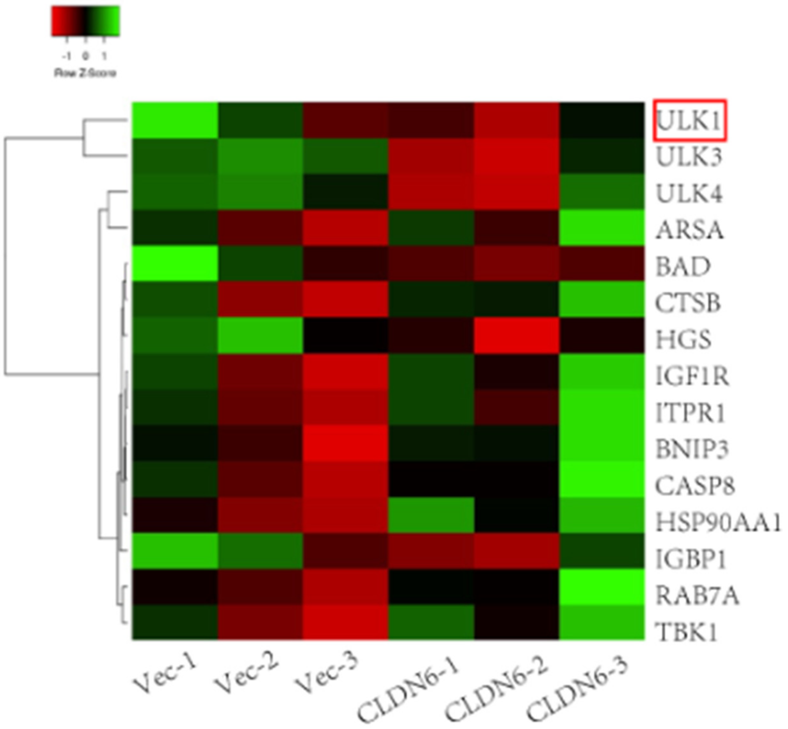

Figure S2. Heatmap of autophagy-related differential genes in MCF-7/CLDN6 cells

Figure S3

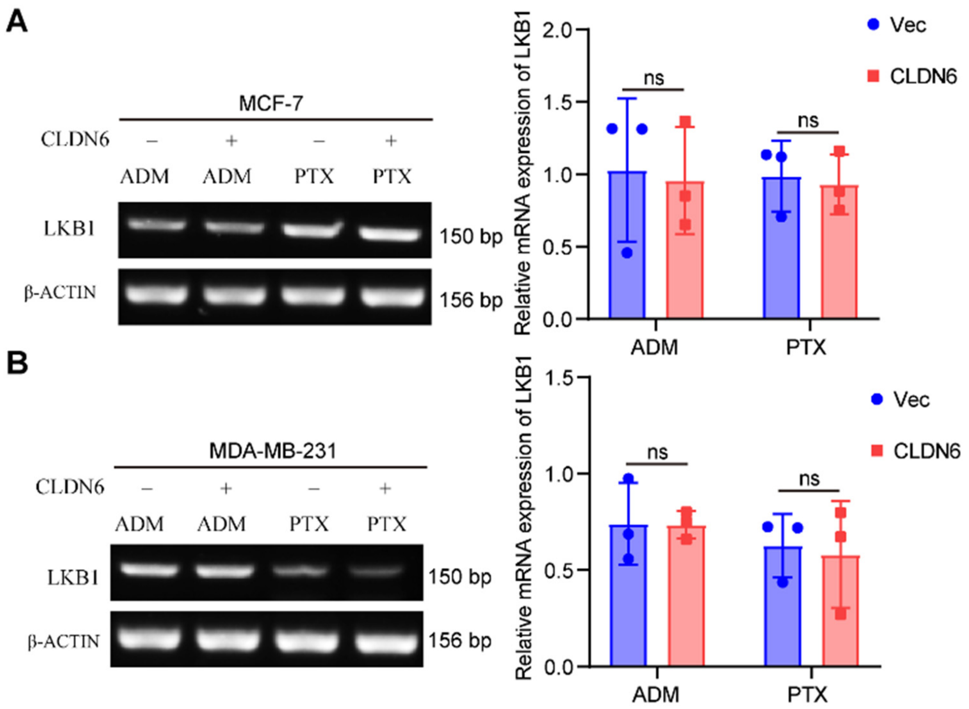

Figure S3. Effect of CLDN6 on mRNA expression of LKB1 under chemotherapy conditions
(A) RT-PCR to detect the effect of CLDN6 on mRNA expression of LKB1 in MCF-7 cells under ADM and PTX treatment; (B) RT-PCR to detect the effect of CLDN6 on mRNA expression of LKB1 in MDA-MB-231 cells under ADM and PTX treatment. (n=3). ns, no significance.

Figure S4

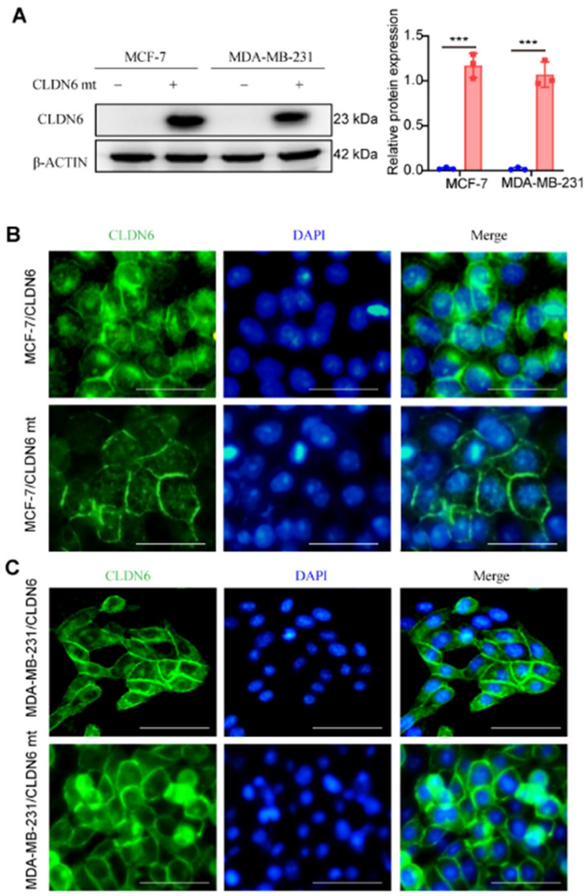

Figure S4. Construction and characterization of breast cancer cells overexpressing CLDN6 mt

(A) WB to detect the protein expression of CLDN6; (B) IF to detect the subcellular localization of CLDN6 and CLDN6 mt. (n=3). Scale bar, 50 μm. ***P < 0.001.

Figure S5

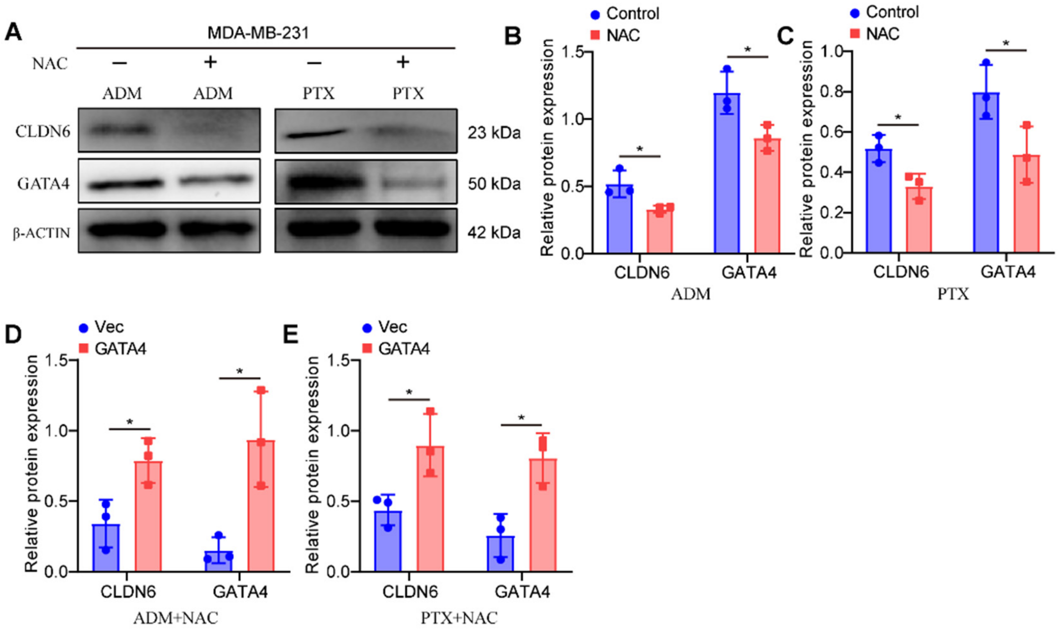

Figure S5. Effect of NAC and GATA4 on the protein expression of CLDN6 and GATA4 under chemotherapeutic conditions

(A-C) WB to detect the effect of NAC on the protein expression of CLDN6 and GATA4 under ADM and PTX; (D-E) WB statistics of the effect of GATA4 overexpression on the protein expression of CLDN6 and GATA4 under NAC and ADM or PTX. (n=3). *$P < 0.05$.

TABLE S1 Details of the antibodies and reagents used in this study

Antibodies or reagents	Information
CLDN6	Santa Cruz Biotechnology Cat# sc-393671
AMPK	Santa Cruz Biotechnology Cat# sc-74461
LKB1	Santa Cruz Biotechnology Cat# sc-32245
GATA4	Proteintech Cat# 19530-1-AP
β-ACTIN	Proteintech Cat# 66009-1-Ig
p62	Proteintech Cat# 18420-1-AP
p-ULK1	Cell Signaling Technology Cat# 5869
p-AMPK	Cell Signaling Technology Cat# 50081
ULK1	Affinity Biosciences Cat# DF7588
NAC	Selleck
ADM	Selleck
PTX	Selleck
CQ	MCE
3-MA	MCE
Baf-A1	MCE
CHX	MCE

TABLE S2 The primers used in RT-PCR

Genes	**Primer Sequence**
β-ACTIN	5'-TCATGAAGTGTGACGTGGACATC-3'<br>5'-CAGGAGGAGCAATGATCTTGATCT-3'
CLDN6	5'-TTCATCGGCAACAGCATCGT-3'<br>5'-GGTTATAGAAGTCCCGGATGA-3'
GATA4	5'-GACTTCTCAGAAGGCAGAGAGTG-3'<br>5'-GATGCCGTTCATCTTGTGGTAGA-3'
LKB1	5'-GCTGGTGGATGTGTTATACAACG-3'<br>5'-TCAATCAGCTGACAGAAGTACCC-3'
CLDN6(ChIP)	5'-CCTGGGTGACACAGTGAAAATA-3'<br>5'-CGTTTCCTACTCTGCAAAATGGAG-3'
